# Comparison of the Effect of Alpha and Hydrocortisone Ointments on Prevention of Acute Skin Complications Due to Radiotherapy in Breast Cancer Patients

**DOI:** 10.1155/2021/5575688

**Published:** 2021-06-16

**Authors:** Mansour Rezaei, Ahmad Khoshay, Nasrin Amirifard, Ali Goli, Alireza Abdi

**Affiliations:** ^1^Department of Biostatistics, Social Development and Health Promotion Research Center, Kermanshah University of Medical Sciences, Kermanshah, Iran; ^2^Department of Nursing, School of Nursing and Midwifery, Kermanshah University of Medical Sciences, Kermanshah, Iran; ^3^Department of Internal Medicine, Faculty of Medicine, Imam Reza Hospital, Kermanshah University of Medical Sciences, Kermanshah, Iran; ^4^Department of Nursing, Student Research Committee, School of Nursing and Midwifery, Kermanshah University of Medical Sciences, Kermanshah, Iran

## Abstract

**Background:**

Radiotherapy in breast cancer patients is associated with acute and delayed side effects. This study aimed to compare the effect of alpha and hydrocortisone 1% (H1%) ointments on prevention of acute skin complications due to radiotherapy in breast cancer patients.

**Methods:**

This clinical trial was conducted on 86 patients with breast cancer in the radiotherapy center of Imam Reza Hospital of Kermanshah, Iran. Using the records, the patients were selected and randomly divided into alpha and H1% groups after obtaining informed consent. The severity of dermatitis, complications, and patient complaints during treatment were evaluated weekly for up to 6 weeks by RTOG criteria. Data were analyzed using SPSS-16 software.

**Results:**

At the end of the third, fourth, fifth, and sixth weeks, 10 (11.7%), 25 (29.1%), 53 (61.6%), and 28 (32.6%) patients had skin complications, respectively. In weeks 5 and 6 in the H1 group, the incidence of complications was higher (*P* = 0.001). The frequency of pain and burning complaints at the end of the third, fourth, fifth, and sixth weeks was 15 (17.4%), 37 (43.0%), 52 (60.5%), and 1(1.2%), respectively. Pain and burning intensity in the fourth and fifth weeks in the H1 group was lower than alpha (*P* = 0010). Complaints of skin itching at the end of the third, fourth, and fifth weeks were 16 (18.6%), 25 (29.1%), and 28 (32.6), respectively. This complication was lower in the H1% group during these weeks (*P* < 0.05).

**Conclusion:**

Alpha ointment is more effective than H1% in relieving pain and burning, preventing complications except itching. It seems using an alpha ointment or combining it with H1% is an appropriate strategy to reduce the rate of injuries and skin complications of radiotherapy.

## 1. Background

Breast cancer is one of the most common cancers, and its increasing rate in recent years has made it one of the most common malignant diseases among Iranian women [1]. Nowadays, due to the expansion of the use of diagnostic facilities such as mammography and increased medical services, breast cancer is often diagnosed in the early stages, but no significant reduction in mortality has been observed. The common age of this disease in Iran is between 35 and 45 years [2]. Breast cancer is the most common cancer and the second leading cause of cancer death in women. During 2007–2009 in Iran, 212,266 cases of cancer were registered in the country's cancer registry system. The total number of breast cancer patients among women was 21,982 (21.7% of all women with cancer) during these years. In 2009, the number of breast cancer patients in Kermanshah was 164 out of 781 cases of women's cancer. In 2014, 232,670 new cases of breast cancer were recorded in women and 2,360 in men in the United States, including 14% of all cancer cases [3].

Breast cancer treatment includes a variety of methods such as radiotherapy. External radiotherapy with External Beam Radiation Therapy (EBRT) radiation is a common treatment for half of the cancer patients [4]. Radiation therapy uses a special type of energy (ionizing radiation) to kill cancer cells. Radiotherapy destroys or damages cells in the target tissue by flawing their genetic nature and does not allow them to grow or divide. Although radiotherapy damages both cancer cells and normal cells, most natural cells recover from radiation therapy effects and recover their normal function. The aim of radiotherapy is to further destroy cancer cells and to inhibit or reduce damage to adjacent healthy tissues [5].

The use of radiotherapy is to reduce the growth or eradication of cancer cells that achieve the desired therapeutic results using ionizing-radiation-producing devices and shining it on the tumor site with doses appropriate to the type of cancer, its location and size, age, and general condition [6, 7]. The purpose of radiotherapy as a topical treatment alone or along with other common treatments is to provide the maximum benefit and minimum side effects [8, 9]. Ionizing radiation (photon, gamma, X-ray, etc.) ionizes the cell's DNA molecules, which prevents cell proliferation and rereplication and, ultimately, cell death [10].

Since almost all rays pass through the patient's skin, skin complications usually take place at the site of radiation entry and sometimes at the exit site [11]. Radiotherapy is associated with acute and delayed side effects that changes in skin tissue health. Approximately 90% of patients receiving radiotherapy experience acute skin complications at the treatment site during the first 1–5 weeks of treatment. Skin complications range from local itching, redness, dry skin, blisters, dry scaling to moisturized scaling, ulcers, and skin necrosis [12]. The incidence and severity of skin complications depend on the factors associated with the treatment method such as energy, dosage, use of radiation bolus volume, length and place of treatment, and use of concomitant treatments such as chemotherapy, as well as individual factors [13]. Skin complications cause physical and mental discomfort, decrease in quality of life, decrease in self-esteem, impaired body image, prolongation, incomplete treatment, increased medical costs, and absenteeism and impose high costs on the health system [14, 15].

Areas of the body such as the groin and armpit are at a higher risk of skin complications due to the effect of bolus (absorbing higher doses of radiation) [16]. The Radiation Therapy Oncology Group (RTOG) is the most common criterion for skin complications that uses in 65% of experimental studies to assess skin complications [17]. The first stage of skin reaction is to reduce sweating and then redness and rash due to capillary dilatation in response to inflammation caused by baseline cell damage [18]. Skin rashes start during the first 3-2 weeks of treatment [19]. Thickening of the layer of the skin and dry scaling occurs in the next step, resulting from an increase in the number of dead cells and is associated with pain and occurs during the third week [20]. In the next step, which can be seen in some patients, scaling is wet and occurs when the base layer produces insufficient cells to replace the lost cells, leading to the destruction of the epidermis and the falling out of the pain [21]. At this point, the skin appears red, bright, and swollen and can develop blisters or ulcers and is at risk of infection [20]. About 45% of breast cancer patients have wet scaling [22]. Macmillan et al. [23], Porock and Kristjanson [6], and Fisher et al. [24] expressed wet scaling in these patients 28%, less than 10%, and 3%, respectively. Wet scaling may lead to discontinuation of treatment due to the necessity of restoration opportunity to normal cells and consequently provide the ground for the return of cancer cells [25]. Barkham announced that 87% of centers stop treatment in case of wet scaling [26]. But, Lavery shows that this is only 30% [27].

The end stage is skin necrosis complications in which the skin becomes dark and leathery [28]. Currently, due to the existence of modern advanced radiotherapy devices, this complication is rarely observed [26]. Many studies have not been conducted to determine the prevalence of acute skin complications in Iran. In Jalilian and Arbabi's study, none of the patients showed grade 4 burn, 31.5% of the patients had grade 1, 64.5% grade 2, and 4% grade 3 [29].

Today, there are no treatment guidelines for the prevention and treatment of skin complications of radiotherapy, and each center has its own guidelines. Most centers do not take preventive measures in terms of the use of topical drugs except for observing the minimum hygienic standards and washing the skin with lukewarm water and mild soap [30, 31]. Proper treatment and the preventive regimen should provide a soft, moist, and sufficient oxygenation environment to control inflammation and prevent skin complications [12].

The high incidence of skin complications of radiotherapy has been a stimulant for effective preventive methods for the treatment of complications. In general, methods such as moisturizing the skin of the treated area and using protective creams and corticosteroids, aloe vera, and other hydrophilic products without lanolin have been recommended [32].

Breast cancer may involve different breast tissues, lymph nodes, and adjacent tissues or metastasis to other parts of the body [33]. Interventions such as the use of herbal medicines such as calendula ointment, aloe vera gel, henna compounds, topical corticosteroid compounds such as cutaneous hydrocortisone, betamethasone, and dexpanthenol, hyaluronic compounds, and mometasone have been conducted in some radiotherapy centers to prevent or treat skin complications of radiotherapy in a clinical trial [34]. Regarding a lack of research, this study was aimed to compare the effect of alpha ointment and 1% hydrocortisone ointment on the prevention of acute skin complications due to radiotherapy in breast cancer patients.

## 2. Methods

This double-blind randomized clinical trial was conducted after obtaining the approval of the research deputy and ethics committee of Kermanshah University of Medical Sciences and obtaining IRCT code with irct2015052114333n34 on the Iranian Clinical Trials website. Samples were selected from patients with breast cancer referred to the radiotherapy center of Imam Reza Hospital in 2015 who had the inclusion criteria and signed informed and written consent to participate in the study. In this study, the patients with grade 2–4 breast cancer who underwent radiotherapy were selected by convenience sampling and randomly assigned to two groups of intervention.

With the permission of the hospital officials and offering information about the radiotherapy department, the researchers referred to the radiotherapy department and provided face-to-face instructions to patients on how to take care of the skin and the application of ointments. Patients in two groups received alpha ointment (Alpha Development company product) and 1% hydrocortisone ointment (Sina Darou product) from the first day of radiotherapy to one week after treatment. In both groups, at least two hours after the first session of radiotherapy, patients used a thin layer of ointment (1.5 cm of ointment length per 100 cm^2^ of skin) on the radiotherapy site, and 8 hours after the first session, they used the ointment again by the mentioned method. Every morning before radiotherapy, as well as on Thursdays and Fridays, they washed the place with lukewarm water and mild baby soap so that no ointment was left on the skin during radiotherapy. The ointments were used twice a day for five days and once for two days (the days received radiation, using the drug was carried out one time). Patients received radiation between 45 and 55 Gray during treatment, receiving an average of 5 weeks and two sessions per week in divided doses. To prevent bias, medications are delivered to patients in coded containers. The examiner's physician and patients were not aware of the contents of the dishes (double-blind). In Imam Reza Hospital, a linear accelerator/Linac is currently used for external beam radiation.

Using Ansari et al.'s [25] study and based on the formula for calculating sample size for comparing the mean of two groups with 95% confidence and 90% power, a total of 82 breast cancer patients referred to the radiotherapy center of Imam Reza Hospital (41 patients in each group) were included in the study. Considering the 10% fall in samples, 90 samples were selected and entered into the study. Of these 90 patients, 4 were left out of the study due to lack of cooperation and lack of medications and care instructions. Finally, 43 patients in each group and a total of 86 patients were studied. Inclusion and exclusion criteria were determined according to relevant studies and similar internal and external articles [2, 3, 8, 9, 16]. They were as follows: diagnosis of advanced or local breast cancer pathology, being treated with a definite or modified mastectomy, undergoing radiotherapy, observing the lack of concurrent chemotherapy, no previous radiotherapy history, no diabetes, no burn wounds, infections, and previous skin complications at the radiotherapy site, no vascular and connective tissue disorders, and signing a consent form approved by the ethics committee. Exclusion criteria included the following: reluctance of the patient to continue participating in the project or discontinuation of cooperation, the history of collagen and vascular or diabetes diseases in the patient during treatment, taking any medication that interferes with wound healing and skin health (such as using systemic steroids during treatment), intolerance or stopping radiotherapy for any reason, and death of the patient.

These two methods were not used alone in previous studies. However, similar to this method and the use of drugs used in this study, it was found that the drugs used had no side effects on patients' skin. In this study, patients referred to the radiotherapy department of Imam Reza Hospital in Kermanshah to complete treatment and eradication of cancer cells and received 5 days per week radiation from linear accelerator to meet their needs and between 25 and 30 sessions.

After the intervention, the data were evaluated by a radiotherapist every week using the criteria for grading skin complications due to radiotherapy (RTOG) as observation and severity of complications such as pain, burning, and itching of the skin based on the patients' announcement and feeling and the findings were recorded in the relevant forms. Acute skin complications are a set of complications such as pain, burning, itching, edema, scaling, and scarring in the skin of the area under radiation therapy, which occurs from a few hours to a few weeks after the onset of radiotherapy [6, 15]. In evaluating skin surface changes based on RTOG criteria, scores change from zero to four. In this way, “zero” is used for no change in the skin, “one” for dry scaling and reduction of sweating and rush occurrence, “two” for painful erythema (redness) and wet scaling of patches and moderate swelling, “three” for wet and clear and intense scaling and pitting edema, and finally, “four” for wounds, necrosis, and bleeding. The criterion for pain, burning, and pruritus in this study is the score that is recorded from 0 to 3 according to the patients' responses. Patients' complaints about pain and skin irritation and itching at the treatment site for the response (in no way, low, moderate, and high) were considered as scores (0, 1, 2, and 3), respectively.

Demographic data, radiation level, and the number of radiotherapy sessions were recorded in separate forms. Details and radiotherapy programs including type and amount of radiation, number of sessions, and radiation fields were also recorded in patients' records. Data included age, date of marriage, marital status, number of children, disease (diagnosis), employment status, life location (city-village), history of the disease and skin allergy, history of diabetes and connective vascular disorders, history of rheumatism, history of previous surgery or radiotherapy in the chest and recent radiotherapy site, history of breast cancer surgery, last date of chemotherapy, the date of the first day of radiotherapy, the overall dose of radiation, and the number of radiotherapy sessions.

Data were analyzed by SPSS-16 software using chi-square, Fisher's exact test, the Kolmogorov–Smirnov test, an independent *t*-test, Lon, and Mann–Whitney *U* tests. The only limitation of possible noncompliance with the method and time of application of ointments by some patients was due to low literacy and lack of understanding of the guidelines, which was explained to them in a simple way via local language to reduce this probability. Also, the first-degree relatives of the patients who were always beside the patient were educated.

In terms of ethical considerations, the participation of all patients in this study was carried out with the written consent of the patients, and the patients' privacy from design to treatment, application training, and weekly examinations were observed. All patients participating in the study were assured that their information remained confidential.

## 3. Results

The Kolmogorov–Smirnov test showed that the variables of age and the distance between surgery and radiotherapy had a normal distribution and the variables of the number of children and marriage age had nonnormal distribution. There was no significant difference between the two groups about average age (*P*=0.621), the interval between surgery and the onset of radiotherapy (*P*=0.885), and the distance between chemotherapy and radiotherapy (*P*=0.068). The highest frequency of samples was in the age range of 40 to 49 years. The minimum age of marriage was 15, and the maximum age of marriage was 37 years. The highest frequency of the samples was in the range of marriage age of 15–24 years (Table 1). There was no significant difference between the two groups in the mean age of marriage (*P*=0.084) and the mean number of children (*P*=0.219).

Most of the samples were married in both groups. The most common samples were 69 (80.2%) who were residents of the city. There was no statistically significant difference between the two groups in terms of location (*P*=0.176). The highest frequency of education level in all samples belonged to the illiterate with 21 (24.4%). The study of the status of the samples in terms of employment showed that the highest frequency of housewives was 75 (87.2%) and 11 (12.8%) were employees (*P*=0.33) (Table 2).

The highest frequency was related to 50 Gray doses (82.6%), and there was no statistically significant difference between the two groups in terms of the total dose of radiation. The highest frequency of the number of sessions (82.6%), 25 sessions in all samples, and both groups was not significant. Radical surgery was the most common in both groups. There was no significant difference between the two groups in terms of surgical procedures (*P*=0.097) (Table 3).

In the first week, no complications were observed in any of the patients based on RTOG criteria. At the end of the second week, only one patient had skin complications from grade 1 and as a rash in the old breast area. In the third week, 10 (11.7%) patients had skin complications. The alpha ointment was more effective than hydrocortisone ointment in preventing skin complications in the third week. In the fourth week, 25 (29.1%) had skin complications. The incidence of complications in the hydrocortisone group (23.3%) was more than alpha ointment (*P*=0.001).

**Figure 1:**
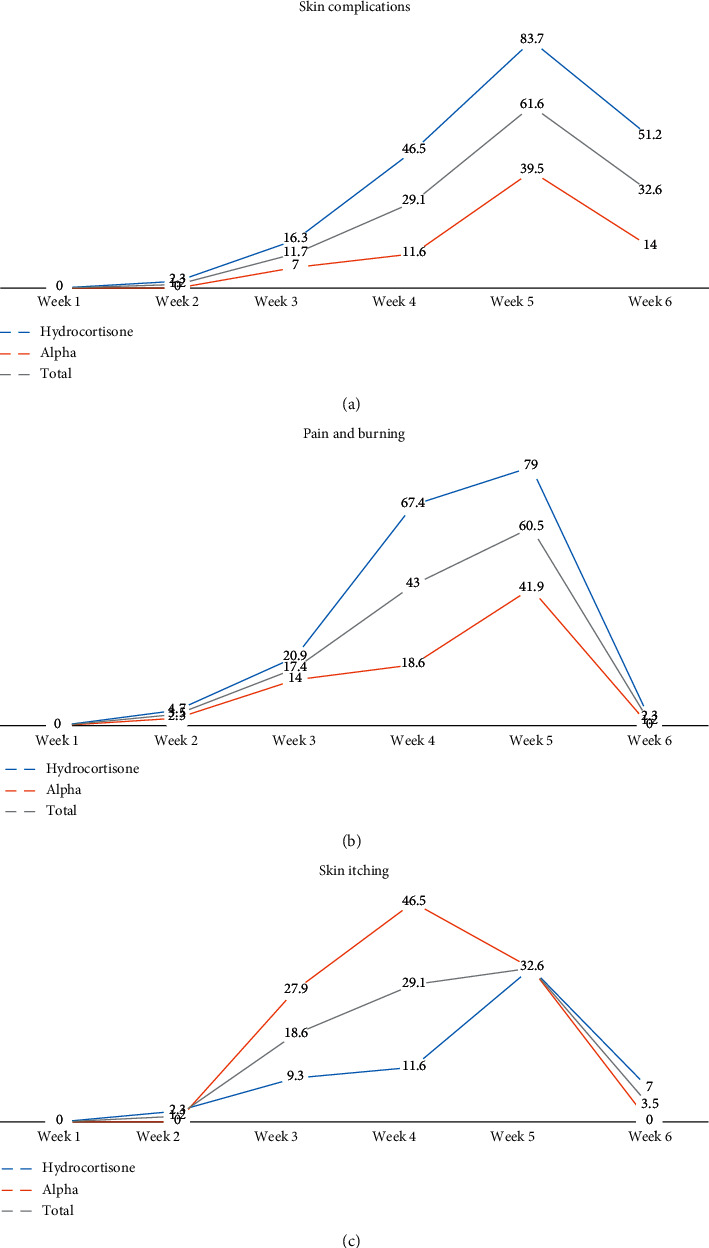
The percent of skin complications, pain and burning, and skin itching.

In the fifth week, 53 (61.6%) had skin complications. The highest frequency of complications at the end of the fifth week was in the hydrocortisone group (36 patients) (41.8%) (*P* < 0.001). In the sixth week, 28 (32.6%) had skin complications. The highest frequency of complications at the end of the sixth week was in the hydrocortisone group (22 patients) (25.5%). In the last week, the alpha ointment was more effective than hydrocortisone ointment in preventing complications (*P* < 0.001) (Table 4 and Figure 1).

In the first and second weeks, pain and burning rate has no difference between the two groups. At the end of the third week, the frequency of complaints of pain and burning was 15 (17.4%), nine in the hydrocortisone group and six in alpha (*P*=0.394). At the end of the fourth week, the frequency of complaints of pain and burning totaled 37 (43.0%), and in the hydrocortisone group, it was 29 (33.7%). At the end of the fourth week, taking alpha ointment with a frequency of 8 was more effective than hydrocortisone ointment with a frequency of 29 in pain relief and burning (*P* < 0.001). At the end of the fifth week, the frequency of complaints of pain and burning totaled 52 (60.5%). The highest frequency was related to the hydrocortisone group (34 patients) (39.5%). The most complaints of pain and burning in the two groups in the fifth week were mild (low), and one complaint of moderate pain was available in the hydrocortisone group. At the end of the fifth week, taking alpha ointment with a frequency of 18 was more effective than hydrocortisone ointment with frequency 34 in pain relief and burning (*P*=0.001). The frequency of complaints of pain and burning in the sixth week was 12 (14.0%). The most complaints were related to the hydrocortisone group (9 patients) (10.5%). The maximum pain and burning intensity in the two groups in the sixth week was also declared mild (low) (*P*=0.0620) (Table 4).

In the first week, none of the patients complained of skin itching. At the end of the second week, the frequency of skin rash complaints in all samples was 1 case (1.2%) in the hydrocortisone group. At the end of the third week, the frequency of this complaint in all samples was 16 (18.6%), and the highest frequency was related to the alpha group (12 patients) (13.9%) (*P*=0.027). Complaints of skin itching in all samples in the fourth week were 25 cases (29.1%). The highest frequency was recorded in the alpha group 15 (17.5%) (*P*=0.001). Complaints of skin itching in the fifth week of all samples were 28 cases (32.6%) which recorded that, in both groups, the frequency was the same and its severity was mild (low) and at the end of the sixth week, the frequency in all samples was 3 cases (3.5%) that belonged to the hydrocortisone group (*P*=0.078) (Table 4). It seems that hydrocortisone ointment is more effective than alpha ointment in preventing and relieving skin itching.

## 4. Discussion

The results showed that no complications were observed in any of the patients in the first week. In the fourth week, 25 (29.1%) skin complications (hydrocortisone group with 20 patients four times that of the alpha group) were observed. In the fifth week, skin complications were higher in the hydrocortisone group. The sixth week was almost the same. Therefore, the alpha ointment was more effective than hydrocortisone ointment in preventing complications.

In terms of pain and burning at the end of the fourth week, the frequency of complaints of pain and burning in all samples was 37 cases (hydrocortisone group was approximately 3.5 times alpha). At the end of the fifth week, it was also lower in the alpha group. At the end of the third, fourth, and fifth weeks, hydrocortisone ointment was more effective than alpha ointment in preventing and relieving skin rash. At the end of the sixth week, 82.6% of the samples were not radiotherapy (patients with 25 sessions of radiotherapy), so the incidence of skin complications declined compared to the fifth week. The process of complaint of pain, burning, and itching of the skin also declined, indicating the effect of interventions on reducing complications and relieving pain and itching of the skin of patients. However, the effect of radiation cutting and end of treatment can also be due to it.

The importance of skincare and prevention of skin complications due to radiotherapy is an important nursing issue. Nursing is protecting, promoting, and improving health and ability, preventing disease and injuries, reducing pain and suffering through diagnosis and treatment of human responses, and defending the caring rights of individuals, families, communities, and communities [34, 35].

In other studies, exactly these two drugs have not been compared. However, some studies are used to familiarize them with their results and compare their results with the present study. Bostrom et al. in a clinical trial study compared the use of mometasone furoate ointment and softening cream on patients with breast cancer undergoing radiotherapy, and skin erythema was 25% and 60%, respectively [36]. In one study, Schmuth et al. compared the effects of dexpanthenol 5% cream and methylprednisolone spoonate 1% cream, which did not affect reducing skin complications. Even prednisolone consumption caused severe skin reactions [37]. Shukla et al. examined the use of beclomethasone spray in a study on the prevention of wet scaling (in 13% of patients in the study group) compared to the nonuse of other corticosteroids (in 37% of the patients studied) [38]. In a clinical study conducted by Farhan et al. on 76 cancer patients and compared betamethasone ointment with placebo to prevent acute radiotherapy dermatitis, the results showed that betamethasone ointment reduces skin complications of radiotherapy [39].

In their review study entitled “History of Agriculture, Consumption, Ecology, and Geographical Distribution of Henna,” Kumar et al. considered the use of henna in previous eras as common for dyeing skin, hair, and nails and declared that henna has antifungal and softening properties of the skin [40]. In the alpha ointment used in the present study, henna is used and confirms the results of this study in coordination with the abovementioned properties. Nadkarani confirmed that henna has antifungal, anti-inflammatory, analgesic, and soft skin moisturizing properties [41]. Hosseini et al. also concluded a study entitled “Comparing the effect of alpha ointment and silver sulfadiazine in the treatment of Pseudomonas infection in third-degree burns” on mice and concluded that wound infection was significantly lower in the alpha consumer group [42].

Omidvari et al. conducted a clinical trial entitled “The effect of topical honey, hydrocortisone ointment, and simple washing on the improvement of radiotherapy-induced dermatitis in breast cancer patients” in Namazi Hospital in Shiraz in 2008. They concluded hydrocortisone significantly improved the severity of patients' symptoms such as burning and itching compared to honey [43]. The results of this study are similar in terms of reducing pruritus.

Fotouhi et al. conducted a study entitled “Comparing the effect of calendula ointment and betamethasone in the prevention of acute radiation dermatitis” in Imam Khomeini Hospital in Tehran during 2004-2005. The results of this study showed that calendula ointment has an effect on reducing the severity of acute dermatitis and, at the same time, has no long-term side effects [44].

A clinical trial study titled “Comparison of grade 2 burn healing time in two methods of dressing with Plant Ointment of fundermol (Alpha) and Silver Sulfadiazine Ointment 1%,” in 2010, of patients with grade 2 burns with a level of between 1 and 10%, age 2–60 years, time of referring to the hospital less than 6 hours after the occurrence of burns, and thermal burns with hot liquids or objects was performed in Imam Musa Kazem Hospital in Isfahan. The duration of burn wound healing in the group treated with fundermol ointment was shorter than that in silver sulfadiazine group 1, and the fundermol ointment was more effective than silver sulfadiazine 1% and relieved the pain in patients. Also, fundermol treated infection better and was more economically cost effective [45]. In terms of reducing pain, the results of this study are similar to those of the current study.

A clinical trial was conducted by Nawab et al. at Ajmal Khan Medical School Hospital in Uttar Pradesh, India, during 2001–2003 under the title of clinical effects of Unani formulation on eczema treatment. The formulation consisted of henna extract, black bean extract, and olive oil which was prepared in the laboratory of the Faculty of Pharmacy. 30 patients with severe eczema with skin symptoms such as itching, burning, redness, and erythema, popular edema, and lesions were selected. The results indicated that henna-containing compounds had a positive effect on improving the signs and symptoms of eczema [46]. These results are in line with the findings of the current study.

A randomized clinical trial was conducted in 2001 by Leigh Olsen, and his work aimed at whether the use of aloe vera gel and mild soap would reduce skin reactions in patients undergoing radiation therapy compared to mild soap alone. The results of this study indicated that aloe vera gel was allergic in some people, but it was useful for most people to protect the skin from radiation. Therefore, in people susceptible to skin problems, aloe vera can somehow increase their tolerance [47].

A randomized clinical trial, in 2013 by Ansari et al., on the efficacy of alpha topical ointment (containing natural henna extract) in comparison with cutaneous hydrocortisone 1% in the treatment of radiotherapy-induced dermatitis in breast cancer patients was conducted on 60 breast cancer patients undergoing radiotherapy at Namazi Hospital in Shiraz. 60 patients who had grade 2 and 3 dermatitis after breast cancer radiotherapy and receiving 45–50 Gray radiation were selected. All patients had mastectomy and chemotherapy before radiotherapy. The results showed that the use of alpha ointment was significant in improving skin complications and radiotherapy-induced dermatitis in breast cancer patients compared to hydrocortisone ointment. It was also more effective in reducing patients' complaints of pain, pruritus, and skin edema than hydrocortisone. However, there was no difference in skin irritation reduction in the two groups [48]. The results of this study are against the results of this study in terms of pruritus, but they are similar in other cases, perhaps the cause of conflict in elective patients.

This clinical trial was conducted by Hemati et al. entitled “Silver Sulfadiazine 1% Ointment in Prevention of Radiation-Therapy-Induced Dermatitis in Breast Cancer Patients” in Isfahan Seyed-al-Shohada Hospital in 2009-2010. The results of this study showed that silver sulfadiazine ointment has a positive effect on reducing acute skin complications and relieving pain and its severity [49].

Radiotherapy as a usual treatment is also used for other skin cancers, such as nonmelanoma skin cancer [50] and malignancies [51, 52]. However, almost all of them, acute and chronic side effects, e.g., dermatitis, erythema, ulceration, and fibrosis, will occur. Because there are no consensus treatments for these side effects [53] and due to lack of studies on this issue, the components of this study, hydrocortisone and alpha ointments, would be beneficial, which demanded more investigations.

Studies and different methods in which they have been studied show different results. Most of the studies are extratherapeutic, and preventive studies are different in terms of the type of drug and intervention used. It is hoped that, by doing this plan, we have taken a small step in choosing a better method of skincare in order to reduce the complications of radiotherapy, increase the quality of treatment, and ultimately, patients' satisfaction with treatment.

## 5. Conclusions

In this study, in the hydrocortisone group, the incidence of skin complications was higher, the severity of pain and burning was lower, and complaints of skin itching were lower than in the alpha group. Therefore, alpha ointment is more effective than hydrocortisone ointment in pain relief and burning, preventing complications except itching. The suggestion of using alpha ointment or combining it with hydrocortisone is a suitable way to reduce the amount of damages and skin complications of radiation therapy and to improve the quality of life of patients. Considering the importance of effective and low-complication treatment in cancer patients, it is expected that the positive results obtained in this study will be used to prevent skin complications of radiotherapy in these patients.

## Figures and Tables

**Table 1:** Comparing mean and SD and quantitative variables of two groups.

Variable	Alpha ointment group	Hydrocortisone ointment group	Total	*P* value
Age (year)	12.33 ± 49.74	48.51 ± 1065	49.13 ± 11.45	0.621
Marriage age (year)	20.39 ± 4.91	21.5 ± 4.05	20.49 ± 4.51	0.084
Children rate	3.42 ± 1.66	3.05 ± 1.69	3.22 ± 1.67	0.219
Surgery to radiotherapy interval (day)	481.91 ± 48.17	681.62 ± 37.25	581.85 ± 42.81	0.885
Chemotherapy to radiotherapy interval (day)	74.73 ± 14.48	82.44 ± 19.34	87.40 ± 17.32	0.068

**Table 2:** Comparing frequency (percent) of qualitative variables of two groups.

Variable		Alpha	Hydrocortisone	Total	*P* value
Marital status	Married	36 (83.7)	39 (90.7)	75 (87.2)	0.333
	Single	7 (16.3)	4 (9.3)	11 (12.8)	

Habitation	City	37 (86.0)	32 (74.4)	69 (80.2)	0.176
	Village	6 (14.0)	11 (26.5)	17 (19.8)	

Education	Under diploma	30 (69.8)	29 (67.4)	59 (68.6)	0.576
	Diploma and higher	13 (30.2)	14 (32.6)	27 (31.4)	

Occupation	Employee	4 (9.3)	7 (16.3)	11 (12.8)	0.520
	Housekeeper	36 (83.7)	39 (90.7)	75 (87.2)	

Total		43 (100.0)	43 (100.0)	86 (100.0)	—

**Table 3:** Frequency (percent) of procedural variables of two groups.

Variable		Hydrocortisone	Alpha	Total	*P* value
Total radiation dose (gray)	50	33 (76.7)	38 (88.4)	71 (82.6)	0.155
	50.4 and 54	10 (23.3)	5 (11.6)	15 (17.4)	

Radiation dose (gray)	200	34 (79.1)	38 (88.4)	72 (83.7)	0.243
	180	9 (20.9)	5 (11.6)	14 (16.3)	

Number of sessions	25	33 (76.7)	38 (88.4)	71 (82.6)	0.155
	27 and 28	10 (23.3)	5 (11.6)	15 (17.4)	

Surgery method	Radical	40 (93.0)	35 (81.4)	74 (87.1)	0.097
	Lumpectomy	3 (7.0)	8 (18.6)	12 (12.9)	

Total		43 (100)	43 (100)	86 (100)	—

**Table 4:** Comparing two groups in terms of skin complication, pain and burning, and skin itching in various times.

Complication		Severity	Hydrocortisone	Alpha	Total	*P* value
Skin complication	W2	G1	1 (2.3)	0	1 (1.2)	0.314
	W3	G1	6 (14.0)	3 (7.0)	9 (10.5)	0.33
		G2	1 (2.3)	0	1 (1.2)	
	Total		7 (16.3)	3 (7.0)	10 (11.7)	
	W4	G1	15 (34.9)	5 (11.6)	20 (23.3)	0.001
		G2	5 (11.6)	0	5 (5.8)	
	Total		20 (46.5)	5 (11.6)	25 (29.1)	
	W5	G1	27 (62.8)	17 (39.5)	44 (51.1)	<0.001
		G2	9 (20.9)	0	9 (10.5)	
	Total		36 (83.7)	17 (39.5)	53 (61.6)	
	W6	G1	14 (32.6)	6 (14.0)	20 (23.3)	<0.001
		G2	8 (18.6)	0	8 (9.3)	
	Total		22 (51.2)	6 (14.0)	28 (32.6)	

Pain and burning	W2	Mild	2 (4.7)	1 (2.3)	3 (3.5)	0.557
	W3	Mild	9 (20.9)	6 (14.0)	15 (17.4)	0.394
	W4	Mild	29 (67.4)	8 (18.6)	37 (43.0)	<0.001
	W5	Mild	33 (76.7)	18 (41.9)	51 (59.3)	0.001
		Moderate	1 (2.3)	0	1 (1.2)	
	W6	Mild	1 (2.3)	0	1 (1.2)	0.314

Skin itching	W2	Mild	1 (2.3)	0	1 (1.2)	0.314
	W3	Mild	4 (9.3)	12 (27.9)	16 (18.6)	0.027
	W4	Mild	5 (11.6)	15 (34.9)	20 (23.3)	0.001
		Moderate	0	5 (11.6)	5 (5.8)	
	W5	Mild	14 (32.6)	14 (32.6)	28 (32.6)	1.0
	W6	Mild	3 (7.0)	0	3 (3.5)	0.078

W = week, G = grade.

## Data Availability

Data can be obtained by contacting the corresponding author.

## Abbreviations

EBRT: External beam radiation therapy
RTOG: Radiation Therapy Oncology Group.
